# Supplementary material for: Child undernutrition is associated with maternal mental health and other sociodemographic factors in low-income settings in Dhaka, Bangladesh
Source: PLoS One. 2025 May 2;20(5):e0322507. doi: 10.1371/journal.pone.0322507 (PMC12047756; doi:10.1371/journal.pone.0322507)
Supplement: S1 Table — (DOCX) [file pone.0322507.s001.docx]

Supplementary Table 1: Scoring technique for the child feeding index

| **Variables** | **Children 6-8 months** | | **Children 9-23 months** | |
| --- | --- | --- | --- | --- |
|  | **Category** | **Score** | **Category** | **Score** |
| Breastfeeding | Yes | 2 | Yes | 2 |
|  | No | 0 | No | 0 |
| Bottle feeding | Yes | 0 | Yes | 0 |
|  | No | 1 | No | 1 |
| Dietary diversity score during 24-h | 0 food groups | 0 | 0 food groups | 0 |
|  | 1-3 food groups | 1 | 1-3 food groups | 1 |
|  | ≥ 4 food groups | 2 | ≥ 4 food groups | 2 |
| Meal frequency during 24-h | 0 meal/day | 0 | 0 meal/day | 0 |
|  | 1 meal/day | 1 | 1-2 meal/day | 1 |
|  | ≥ 2 meals/day | 2 | ≥ 3 meals/day | 2 |
