# Supplementary material for: Child undernutrition is associated with maternal mental health and other sociodemographic factors in low-income settings in Dhaka, Bangladesh
Source: PLoS One. 2025 May 2;20(5):e0322507. doi: 10.1371/journal.pone.0322507 (PMC12047756; doi:10.1371/journal.pone.0322507)
Supplement: Attachment — Submitted filename: Authors response to reviewers comments (2).docx [file pone.0322507.s004.docx]

**Author’s response to the review comments**

Reviewer #1: Interesting idea of this study, my recommendations are the following:

**Comment:** Lines 22-25 I recommend to write as a single sentence, for example - assessment of malnutrition status and associated characteristics in children.

**Author’s response:** Thank you for your suggestion. We have revised the sentences in Lines 22-25 to present the information concisely in a single sentence, as recommended. The revised sentence now reads: "This study assesses child undernutrition and its associated characteristics, including maternal mental health, in low-income settings in Dhaka, Bangladesh." See the lines 21-22 in the revised manuscript.

**Comment:** I recommend expanding the Introduction section by mentioning the main aspects regarding the mental health of mothers and the risk of associated conditions. Description based on specialized literature of the main mental conditions in breastfeeding mothers.

**Author’s response:** Thank you for your thoughtful comment. We recognize the importance of further elaborating on the mechanisms through which maternal mental health influences child nutrition to strengthen the rationale for our study.

To address this, we have expanded the Introduction section to highlight how maternal depression and anxiety can negatively impact feeding practices, healthcare-seeking behavior, and overall child well-being. Maternal mental health issues can lead to reduced responsiveness in caregiving, lower breastfeeding rates, and poor complementary feeding practices, all of which are critical for a child's growth and development. Additionally, mothers experiencing depression or anxiety may have limited energy, motivation, or confidence to ensure proper hygiene, meal preparation, or timely healthcare visits, increasing the risk of child malnutrition and illness. Please see the lines 65-84 in the revised manuscript.

**Comment:** Line 109 I recommend mentioning the bibliographic index as a number from the bibliographic references, according to the editing rules.

**Author’s response:** Thank you for your suggestion. We have revised Line 109 to include the bibliographic reference number in accordance with the journal's editing rules. See the revision “A prior study conducted in an urban slum region in Bangladesh [9], where the prevalence of common mental disorders was 46.2% and stunting was 44.3%, we assumed a sample power of 50%” in the revised manuscript. See the lines 120-121 in the revised manuscript.

**Comment:** Assessment of Maternal Mental Health – I recommend mentioning the reliability of the questionnaires by calculating Cronbach's alpha regarding the results of this study.

**Author’s response:** Thank you for your valuable suggestion. We have now assessed the reliability of the questionnaires used to measure maternal mental health in our study. The Cronbach’s alpha values were 0.854 for the Generalized Anxiety Disorder 7-Item Scale (GAD-7) and 0.807 for the Patient Health Questionnaire-9 (PHQ-9), indicating good internal consistency. We have incorporated this information in the manuscript accordingly. See the lines 164-165 and 171-172 in the revised manuscript.

**Comment:** Characteristics of the study subject – I recommend that when interpreting the results, the information should not be duplicated with those mentioned in table 1, I recommend clarifications.

**Author’s response:** Thank you for your suggestion. We have revised the interpretation of the study subject characteristics to avoid unnecessary duplication of information already presented in Table 1. Instead of repeating detailed numerical data, we now provide a summarized interpretation while referring readers to Table 1 for specifics. See the lines 239-247 in the revised manuscript.

“Table 1 presents the background characteristics of the study participants. The majority of children were under two years old, and most mothers were younger than 25 years. A significant proportion of mothers were homemakers, had only primary-level education, and belonged to low-income households. More than 80% of households reported some degree of food insecurity. The prevalence of child undernutrition was notable, with stunting being the most common form. More than half of the children had a high child feeding index score, while maternal underweight was observed in a small proportion. Regarding maternal mental health, nearly half of the mothers experienced some level of depression, and anxiety was prevalent among a similar proportion. Further details on these characteristics are provided in Table 1.”

**Comment:** I recommend that in tables regarding the p-value index, the standard symbol should be mentioned: <, >.

**Author’s response**: Thank you for your suggestion. The p-value index has been corrected according to your suggestion in the tables.

**Comment:** I recommend that future research directions and practical implications be mentioned at the end of the Discussions section.

**Author’s response:** Thank you for your insightful suggestion. We have now included a section at the end of the Discussion to highlight future research directions and implications. See the following section in the revised manuscript (Lines 426-441).

Future research directions and implications

This study highlights the association between maternal mental health and child undernutrition, emphasizing the need for further research to explore causal relationships. Future studies should adopt longitudinal designs to determine how maternal depression and anxiety influence child growth over time. Additionally, qualitative research could provide insights into how maternal mental health affects child-feeding practices and nutritional outcomes.

From a policy perspective, integrating maternal mental health support into existing maternal and child health programs is essential. Routine screening for depression and anxiety during antenatal and postnatal care could help identify at-risk mothers early. Community-based interventions promoting mental health awareness, social support, and nutritional education should be prioritized to improve both maternal well-being and child nutrition. Targeted nutrition programs are also needed to address the high prevalence of child undernutrition. Special attention should be given to vulnerable groups, such as young mothers and female children, who were identified as being at higher risk. A multi-sectoral approach, combining mental health support, improved feeding practices, and social protection measures, could help reduce the burden of undernutrition in low-income urban settings.

Reviewer #2: The manuscript presents a well-executed study on an important public health issue, exploring the relationship between maternal mental health and child undernutrition in low-income settings. The use of validated mental health assessment tools (PHQ-9, GAD-7) and standardized anthropometric measurements strengthens the reliability of the findings. Additionally, the application of multivariate logistic regression and appropriate model validation enhances the robustness of the statistical analysis. The study’s conclusions are well-supported by the data, and the policy implications are clear, emphasizing the need for maternal mental health interventions in child nutrition programs.

**Comment:** However, the manuscript has several areas that require improvement. The writing quality needs refinement, as certain sentences are overly complex, making it difficult to follow key findings. For instance, in the **Discussion section**, some interpretations of the regression results could be rewritten more concisely to improve clarity. Additionally, there is some repetition in how maternal depression and anxiety are discussed across different sections, particularly in the Results and Discussion, which could be streamlined to avoid redundancy. Statistical interpretations should also be made more precise—for example, rather than stating that maternal depression “considerably increases” the odds of stunting, specifying the exact percentage increase based on odds ratios would make the findings more accessible to readers.

**Author’s response:** Thank you for your detailed feedback. We acknowledge the need for improving writing clarity, reducing redundancy, and refining statistical interpretations to enhance readability and precision.

To address these concerns, we have made the following revisions:

- Simplified complex sentences in the Discussion section, ensuring that key findings and interpretations of regression results are presented more concisely.
- Reduced repetition in the Results and Discussion sections, particularly regarding maternal depression and anxiety, by consolidating overlapping points to avoid redundancy.
- Refined statistical interpretations by specifying exact percentage increases in odds based on odds ratios rather than using broad terms like "considerably increases." This enhances precision and makes findings more transparent for readers.

See the lines 291-319, 329-332, 355-360 in the revised manuscript.

**Comment:** Furthermore, the manuscript would benefit from clearer terminology. The terms “malnutrition” and “undernutrition” appear to be used interchangeably, though they have different meanings—malnutrition includes both under- and overnutrition, while this study focuses specifically on undernutrition. Consistently using the correct term would enhance clarity. Similarly, the description of the child feeding index could be expanded slightly to help readers unfamiliar with the methodology understand how scores were calculated.

**Author’s response:** Thank you for your insightful comment. We acknowledge the distinction between "malnutrition" and "undernutrition" and agree that consistent terminology will enhance clarity. Our study specifically focuses on assessing undernutrition in children, including stunting, wasting, and underweight. To accurately reflect this focus, we have carefully revised the manuscript to ensure that "undernutrition" is used appropriately throughout, replacing "malnutrition" where necessary. We appreciate your suggestion, which has helped improve the precision and consistency of our terminology.

We have expanded the description of the child feeding index in the Methods section to provide greater clarity for readers unfamiliar with the methodology. Specifically, we have detailed how the index was constructed, including the key components assessed, the scoring criteria, and the categorization of feeding practices. We have provided this information as a supplementary table as well. This addition enhances the transparency of our methodology and ensures a better understanding of how the scores were derived.

| Variables | Children 6-8 months | | Children 9-23 months | |
| --- | --- | --- | --- | --- |
|  | Category | Score | Category | Score |
| Breastfeeding | Yes | 2 | Yes | 2 |
|  | No | 0 | No | 0 |
| Bottle feeding | Yes | 0 | Yes | 0 |
|  | No | 1 | No | 1 |
| Dietary diversity score during 24-h | 0 food groups | 0 | 0 food groups | 0 |
|  | 1-3 food groups | 1 | 1-3 food groups | 1 |
|  | ≥ 4 food groups | 2 | ≥ 4 food groups | 2 |
| Meal frequency during 24-h | 0 meal/day | 0 | 0 meal/day | 0 |
|  | 1 meal/day | 1 | 1-2 meal/day | 1 |
|  | ≥ 2 meals/day | 2 | ≥ 3 meals/day | 2 |

**Comment:** Overall, this is a valuable study with important findings, but revisions to improve clarity, reduce redundancy, and refine statistical explanations would strengthen the manuscript further.

**Author’s response:** Thank you for your valuable feedback and for recognizing the importance of our study. We appreciate your suggestions to improve clarity, reduce redundancy, and refine statistical explanations to enhance the manuscript.

To address these concerns, we have:

- Refined the writing to improve clarity and ensure a more concise presentation of findings, particularly in the Discussion section.
- Reduced redundancy by streamlining repetitive content, especially in the Results and Discussion sections, to maintain a focused narrative.
- Improved statistical explanations by providing precise interpretations of odds ratios, ensuring that findings are conveyed with clarity and accuracy.
- Clarified the calculation of the child feeding index to help readers unfamiliar with the methodology better understand how the scores were determined.

See the lines 291-319, 329-332, 355-360 in the revised manuscript. Also see the supplementary table 1.

Reviewer #3: The study titled “Child Undernutrition is Associated with Maternal Mental Health and Other Sociodemographic Factors in Low-Income Settings in Dhaka, Bangladesh” by Karim et al. discusses an important topic. However, to further improve their study, I recommend the following edits:

**Comment:** Pages 11-13, Introduction Section: Given that this study focuses on maternal health, it is important for the authors to elaborate further on maternal mental health and its impact on child nutrition. While the introduction mentions maternal mental health as a factor, it does not sufficiently explain the mechanisms through which maternal depression or anxiety may lead to child undernutrition. Adding a few lines on why maternal mental health is crucial—such as its influence on feeding practices, healthcare-seeking behavior, and overall child well-being—would strengthen the argument and provide a clearer rationale for its inclusion in the study, especially in the context of Bangladesh.

**Author’s response:** Thank you for your thoughtful comment. We recognize the importance of further elaborating on the mechanisms through which maternal mental health influences child nutrition to strengthen the rationale for our study.

To address this, we have expanded the Introduction section to highlight how maternal depression and anxiety can negatively impact feeding practices, healthcare-seeking behavior, and overall child well-being. Maternal mental health issues can lead to reduced responsiveness in caregiving, lower breastfeeding rates, and poor complementary feeding practices, all of which are critical for a child's growth and development. Additionally, mothers experiencing depression or anxiety may have limited energy, motivation, or confidence to ensure proper hygiene, meal preparation, or timely healthcare visits, increasing the risk of child malnutrition and illness. Please see the lines 65-84 in the revised manuscript.

**Comment:** Page 12, Lines 78-85: As the authors mention that non-slum low-income areas remain unexplored, they should expand on this point and highlight it more, as it represents the novel aspect of their study. Many studies have already been conducted on maternal health in low- and middle-income countries like Bangladesh, so it is important to explain why non-slum low-income areas need separate attention. Clarifying how this population differs from those previously studied would help strengthen the focus and contribution of this research.

**Author’s response:** Thank you for your insightful comment. We appreciate the opportunity to elaborate on the significance of studying non-slum low-income areas and how this population differs from those previously studied.

To address this, we have expanded the discussion to highlight that while urban slum populations in Bangladesh have been widely studied regarding child undernutrition, non-slum low-income areas remain underexplored despite facing similar economic hardships. These areas house marginalized populations who may not be as visible in policy discussions, as they are not classified as slum dwellers but still experience limited access to healthcare, social safety nets, and nutritional resources. Their living conditions, employment patterns, and access to services differ from both slum residents and middle-income urban populations, necessitating a focused investigation.

Furthermore, our study provides novel insights by examining the association between maternal mental health and child undernutrition in this specific non-slum low-income context, an area that has received little research attention. We have revised the manuscript to emphasize these points more clearly and strengthen the study's contribution.

“Several studies identified the socio-economic determinants of child undernutrition in Bangladesh at national and subnational levels [5–9, 16]. Urban areas in Bangladesh encompass a diverse population in terms of distinct economic disparity and lifestyle, which might affect the nutrition of children [17]. A few studies were also conducted in urban slum areas to explore child undernutrition and its contributing factors [9, 18, 19]. However, the non-slum low-income regions in cities like Dhaka remain unexplored in terms of child undernutrition and their determinants. Unlike slum dwellers, who often rely on government or NGO-led interventions, non-slum low-income families may have limited access to structured support systems, despite experiencing similar economic hardships. Their housing conditions, employment stability, access to healthcare, and food security differ from both slum populations and middle-income urban residents, making them a unique yet overlooked group in child nutrition research. Understanding the specific challenges faced by these communities is crucial for designing targeted interventions that address their nutritional vulnerabilities. Moreover, while studies linking maternal mental health and child undernutrition are scarce, no previous research has specifically examined this relationship in non-slum low-income urban settings.

In the present study, we examined child undernutrition and maternal mental health in low-income settings in Dhaka, Bangladesh. We also assessed the associations of maternal mental health and other background characteristics with child undernutrition. This finding would provide an in-depth understanding of the factors of child undernutrition for policymakers, guiding decisions to prevent child malnutrition.”

See the lines 84-103 in the revised manuscript.

**Comment:** Page 13, Lines 93-99: The study setting and population are well described; however, the inclusion and exclusion criteria for participants are not mentioned. Clarifying these criteria would enhance the transparency and reproducibility of the study. Were there any specific factors that determined participant selection or exclusion?

**Author’s response:** Thank you for your valuable comment. We acknowledge the importance of clearly defining the inclusion and exclusion criteria to enhance the transparency and reproducibility of our study.

To address this, we have now added a dedicated section outlining the inclusion and exclusion criteria in the Methods section. Participants were included if they were lactating mothers with children aged 6–23 months, permanent residents of the selected low-income areas in Dhaka, and the primary caregivers responsible for child feeding and care. Mothers who were not the primary caregivers, had severe illnesses or disabilities, or had children with congenital anomalies or severe chronic illnesses were excluded. See the lines 124-136 in the revised manuscript.

**Inclusion and exclusion criteria**

Participants were eligible for inclusion in the study if they met the following criteria:

•	Lactating mothers with children aged 6–23 months.

•	Permanent residents of the selected low-income areas in Hazaribagh and Mohammadpur thanas (excluding Geneva camp), Dhaka.

•	Primary caregivers responsible for the child's feeding and care.

•	Provided informed consent to participate in the study.

Participants were excluded from the study if they met any of the following criteria:

•	Mothers who were not the primary caregivers of the child.

•	Mothers with severe illnesses or disabilities that could interfere with their ability to provide information.

•	Children with congenital anomalies or severe chronic illnesses that could influence their growth and nutritional status.

**Comment:** Page 14, Lines 119-125: The methodology for anthropometric measurements is unclear. Were these measurements taken by trained professionals, or were they self-reported? Clarifying this would aid in evaluating the accuracy and reliability of the data.

**Author’s response:** Thank you for your insightful comment. We clarify that all anthropometric measurements were taken by trained professionals following standard procedures to ensure accuracy and reliability. Children’s weight was measured using a SECA scale with an accuracy of 0.1 kg, while height/length was measured using a wooden height-length board, calibrated against a standard anthropometric scale, with an accuracy of 0.1 cm.

We have now clarified this in the Methods section to enhance transparency regarding the accuracy and reliability of our anthropometric data.

See the lines 144-151 in the revised manuscript.

**Comment:** Page 15, Lines 127-138: The study provides a clear description of the PHQ-9 and GAD-7 scales, but it is unclear whether they have been validated for use in the Bangladeshi population. Given that cultural differences can influence responses to mental health questionnaires, providing this information would strengthen the study’s validity.

**Author’s response:** Thank you for your thoughtful comment. We agree that the cultural context is an important factor in the validity of mental health questionnaires. To address this, we would like to clarify that both the PHQ-9 and GAD-7 scales have been validated for use in the Bangladeshi population. The questionnaires were translated into Bangla and pre-tested with a sample from the target population to ensure their cultural relevance and clarity.

Furthermore, both scales have demonstrated good internal consistency (Cronbach's alpha values of 0.807 for PHQ-9 and 0.854 for GAD-7) and reliability in this context. They also exhibit strong construct validity, as evidenced by previous studies that support their effectiveness in measuring depression and anxiety in Bangladeshi individuals. We have now included this information in the Methods section to ensure transparency and strengthen the study's validity.

See the lines 158-172, and 333-340 in the revised manuscript.

**Comment:** Page 31, Lines 365-378: The authors imply that socio-cultural preferences for male children lead to better nutrition but do not provide direct evidence. Citing studies on gender-based food distribution would strengthen this claim. Adding references would enhance clarity and support the argument.

**Author’s response:** Thank you for your insightful comment. We acknowledge the need for supporting evidence to strengthen our discussion on gender-based differences in child nutrition. To address this, we have added relevant references on socio-cultural preferences for male children and their impact on food distribution and nutritional outcomes. These citations provide context and support our interpretation of the observed disparities in undernutrition between male and female children. See the lines 383-385 in the revised manuscript.

We have included the following reference.

35. Rahman A. Cultural Norms and Son Preference in Intrahousehold Food Distribution: A Case Study of Two Asian Rural Economies. Rev Income Wealth 2019; 65: 415–461.
